# Buttonhole Cannulation Is Not Associated with More AVF Infections in a Low-Care Satellite Dialysis Unit: A Long-Term Longitudinal Study

**DOI:** 10.1371/journal.pone.0142256

**Published:** 2015-11-17

**Authors:** Clémence Béchade, Tony Goovaerts, Philippe Cougnet, Laura Labriola, Michel Jadoul, Eric Goffin

**Affiliations:** 1 Nephrology, CHU Clemenceau, Caen Cedex, France; 2 Nephrology, Cliniques Universitaires Saint-Luc, Université Catholique de Louvain, Brussels, Belgium; Robert Bosch Hospital, GERMANY

## Abstract

**Background:**

Buttonhole cannulation (BHC) has been associated with a greater risk of arteriovenous fistula (AVF)-related infections and septicemia than the rope ladder cannulation (RLC) in in-center hemodialysis (HD). Such infections have never been studied in satellite HD units.

**Study Design:**

Retrospective single center study.

**Setting and Participant:**

All patients in our satellite HD unit using a native AVF from 1 January, 1990, to 31 December, 2012.

**Study Period:**

Two different kinds of cannulation have been used during the study period: From 1 January, 1990 to 1, January, 1998 RLC was used in the unit (period 1). After 1 January, 1998 onwards, all the patients were switched within 3 months to BHC (period 2).

**Outcomes:**

Three different infectious events were observed during the two periods: local AVF infection, bacteremia, and combined infection. The aim of this study was to evaluate the incidence of AVF-related infections in our low-care HD unit and to determine whether BHC is associated with an increased risk of infection in this population.

**Results:**

162 patients were analyzed; 68 patients participated to period 1 and 115 to period 2. Sixteen infectious events occurred. Incidences of AVF-related infectious events were 0.05 [95% CI, 0.02–0.16] and 0.13/1000 AVF-days [95% CI, 0.0.8–0.23], for period 1 and 2 (p = 0.44) respectively. Recurrence of AVF-related infection was observed only during period 2. Unadjusted incidence rate ratio (IRR) of all infections was 0.39 (95%CI 0.12–1.37). Two complicated infections occurred during the study period: one in period 1 and one in period 2.

**Limitations:**

Observational retrospective single centre study

**Conclusions:**

BHC is not associated with an increased infectious incidence in our HD population from a satellite dialysis unit. In the rare patients with AVF-related infection it seems necessary to change cannulation sites as recurrence of infection might be an event more frequent with BHC.

## Introduction

Buttonhole cannulation (BHC) of an arteriovenous fistula (AVF) was described in 1977 by Twardowski [1]. In contrast with the rope ladder technique, a constant site of cannulation is used at each hemodialysis (HD) session. This technique includes first the creation of a tunnel, using sharp needles for an average of 6 dialysis sessions; after that, blunt needles can be used. Careful disinfection and withdrawal of the scabs formed at the cannulation site are needed, and the track formed is used to guide the needle to access the vessel. This technique is associated with less AVF hematoma [2] and aneurysms’ growth [3]. There is also a decrease in bad sticks [4] and in interventions to maintain the vascular access (VA) patency [3, 5]. BHC has been associated with a higher incidence rate of AVF-related infections and septicemia than the rope ladder technique [2, 6–7]. However, these studies only used incidence rate calculation, so that they can not study the risk of recurrence of infection.

Satellite HD units are HD centers with some particularities due to patients’ characteristics and accordingly, to the medical and nursing team. Medical and nursing team is smaller in satellite units. Patients in satellite dialysis units are younger and have less comorbidities than in-center HD patients [8–9]. Whether the peculiarity of these low-care centers could influence outcomes in HD, and particularly AVF-related infections, currently remains ill-defined. Moreover, access related problem is an important issue in HD patients, even in satellite dialysis patients. It has been shown that AVF related complications remain the main cause of fallback to in-center HD in this population [10].

The aim of this retrospective study was firstly to evaluate the incidence of AVF-related infections in our low-care HD unit and secondly to determine whether BHC is associated with an increased risk of infection.

## Materials and Methods

### Study population

We retrospectively included all patients under maintenance HD by a native AVF from 1 January, 1990 to 31 December, 2012, in our satellite HD unit “the Carpe Diem”, in Brussels.

Briefly, and according to our pre-dialysis education program [8], patients can choose a modality of renal replacement therapy, after medical evaluation, and where they want to have dialysis sessions in case of hemodialysis (in hospital center, at home or in the satellite unit). The low-care dialysis unit is designed for autonomous patients who, after a dedicated training, are able to record their clinical parameters (blood pressure and weight measurements, calculation of ultrafiltration …), prepare heparin syringes, self-prepare dialysis machine for use, wash their arm and prepare skin for AVF cannulation, respectively. Some of them self cannulate the AVF whereas others prefer to be cannulated by the single attending nurse. The staff of the Carpe Diem unit includes one doctor and 10 nurses, and there are 6 patients at a time, with 2 shifts 6 times per week.

### Methods

All patients’ charts were retrospectively reviewed by one of us (CB), and supervised by another member of the team (EG). In case of unclear data, medical records were reviewed by colleagues involved in dialysis sessions (LL, MJ). Patient information was anonymized prior to analysis. The study was approved by the biomedical ethics Committee of the Faculty of Medicine of Université catholique de Louvain.

### Covariates

For each patient, age, gender, cause of ESRD, diabetes mellitus, use of immunosuppressive drugs at HD onset, history of kidney transplantation were recorded.

### Outcomes

We were interested in infectious events related to AVF. We defined three different events. *Local AVF infection* was defined as erythema, edema or pain close to cannulation site, or purulent drainage from cannulation site, without any positive blood culture. *Bacteremia* was defined as positive blood culture due to skin micro-organism (Staphylococcus aureus or Staphylococcus epidermidis) without local AVF signs, and without any alternative source of infection. *Combined infection* was considered as a local AVF infection with positive blood culture due to skin micro-organism. Two successive events in the same patient were counted as different events when the second one occurred more than three weeks after antibiotic withdrawal. A local swab was systematically performed in case of erythema or local purulent discharge. Indications for blood cultures did not change throughout the study period: fever or chills, unusual symptoms such as diarrhea, vomiting, and altered mental status. In accordance with the protocol used in the unit, intravenous cefazolin was used as first-line empirical therapy when an infectious event was suspected; intravenous vancomycin was preferred in patients with known colonization by micro-organisms resistant to first-cephalosporin generation. Antibiotics were usually given for 2 weeks.

### Participation time

Person-Time at risk was calculated as the sum of all AVF-days. Number of AVF-days was calculated for each patient during the study period: days between first AVF cannulation and end of treatment, or end of the study period or loss of AVF. Time in HD with a central catheter was excluded from this calculation.

### AVF cannulation

During the study period, 2 different types of cannulation were used. From 1 January, 1990 to 1, January, 1998 rope-ladder cannulation (RLC) was used in the unit. After skin disinfection, sharp needles were inserted in a different site at each HD session. After 1 January, 1998 onwards, all the patients were progressively switched to BHC within 3 months. Access area was carefully disinfected after soap wash. Then scabs were removed from both cannulation sites. A second disinfection was performed before each cannulation with blunt needles, as previously described [11]. Thus, the study period has been divided in two different periods according to the type of cannulation: period 1 (corresponding to RLC), and period 2 (referring to BHC).

### Statistical analysis

Continuous variables were described as median and interquartile range; categorical variables were described as percentages. Comparative statistics used were chi2 test for categorical variables or Fisher’s exact test for counts less than 5, and Student test for continuous variable.

The incidence of infectious events was defined as a person-time rate, with the numerator equal to the number of observed episodes of infection during the period of interest, standardized to 1,000 AVF-days. The incidence rate was compared between the 2 periods with a Fischer exact test.

When we use an incidence rate calculation, defined as (Number of New Cases) / (Person-Time at Risk), every cases of infection are considered independent, and two infections occurring in the same patient are considered as two independent events. We think that two infectious events in the same patient are not independent events and that some other methodological approaches may be more suitable.

To determine if the type of cannulation was associated with infectious event, we performed a Poisson model on the number of events. Actually, this model is appropriate when the outcome is a count, and can be used for modelling rare event, such as infectious event related to AVF. However, as many patients did not present any infectious event during the period (count = 0), hypothesis required for Poisson model were not respected (mean and variance were not equal, meaning overdispersion). A Poisson model adapted to overexpression of zero has been used: we performed a Zero Inflated Poisson (ZIP) model [12]. ZIP model provides two types of coefficient: for each covariates included in the model, an “excess zero coefficient” and a “count model coefficient” are estimated. The former parameter yields the relative odds of the zero proportions in each group while the latter is the change in the relative number of events between the groups, respectively. Statistical significance level was set at p<0.05.

To strengthen the findings, we conducted a sensitivity analysis. We performed two other ZIP models in two different data sets to know how the coefficients may vary.

The data set used for statistical analysis is available in S1 Dataset.

## Results

### Study population

A total of 162 patients receiving HD by a native AVF in our satellite unit were included. All patients were dialysed 4 hours thrice weekly. As shown in Table 1, median age was 45.4 years, and 14.6% of the patients had a history of renal transplantation. The prevalence of diabetes mellitus was higher in second period but the difference between the two groups was not significantly different. Sixty-eight patients participated to Period 1, and 15 of them were converted to BHC on 1 January, 1998; the others had stopped HD with a functioning AVF in our unit before 1998. During Period 2, 115 patients were available for analysis. Noteworthy, 6 patients hemodialyzed during period 1 resumed HD during period 2 after a renal graft failure.

**Table 1:** Patients’ characteristics.

Covariate	1st period (N = 68)	2nd period (N = 115)	P value
	**Median (IQR)**	**Median (IQR)**	
Age at HD initiation (years)	45,8 (20,4)	46,9 (20,4)	**0,9** ^α^
	**Number (%)**	**Number (%)**	
Sex (M)	39 (57,3)	70 (60,9)	**0,6** ^β^
Underlying nephropathy			**0,03** ^δ^
Diabetic	1 (1,5)	9 (7,8)	
Glomerulonephritis	21 (30,9)	44 (38,2)	
Vascular	4 (5,9)	8 (6,9)	
Polycystic kidney disease	15 (22,0)	22 (19,1)	
Uropathy	1 (1,5)	3 (2,6)	
Chronic interstitial nephritis	17 (25,0)	13 (11,3)	
Other	7 (10,3)	13 (11,3)	
Unknown	2(2,9)	3 (2,6)	
Diabetes	2 (2,9)	11 (9,5)	**0,1** ^β^
Transplantation before HD initiation	10 (14,7)	18 (15,6)	**0,7** ^β^
Immunosuppressive therapy at HD initiation	14 (20,6)	25 (21,7)	**0,9** ^β^

^α^ Student test

^β^ Chi2 test

^δ^ Fisher’s exact test

### Incidence of infection

There were 3 AVF-related infections during Period 1 and 13 during Period 2. The calculated absolute incidence per 1,000 AVF-days was thus: 0.05 and 0.13, respectively (p = 0.44) (Table 2). Unadjusted incidence rate ratio (IRR) was 0.48 (95%CI 0.10–0.43) for local infection, 0.42 (95%CI 0.05–3.78) for combined infection and 0.39 (95%CI 0.12–1.37) for all infections.

**Table 2:** AVF-related infections per period.

Infectious event	Period 1	Period 2	p value^δ^
**Local infection (alone)**			
Number	2	7	
AVF-days	57851	97911	
incidence rate (per 1000 AVF-days)	0.03	0.07	0.7
95%CI	(0.028–0.031)	(0.068–0.071)	
**Bacteremia (alone)**			
Number	0	2	
AVF-days	57851	97911	
incidence rate (per 1000 AVF-days)	0	0.02	0.5
95%CI	-	(0.019–0.020)	
**Combined local infection and bacteremia**			
Number	1	4	
AVF-days	57851	97911	
incidence rate (per 1000 AVF-days)	0.02	0.04	0.6
95%CI	(0.002–0.015)	(0.015–0.109)	
**All infections**			
Number	3	13	
AVF-days	57851	97911	
incidence rate (per 1000 AVF-days)	0.05	0.13	0.44
95%CI	(0.02–0.16)	(0.08–0.23)	

^δ^ Fisher exact test

The causative organism was identified in 6 of the 9 local infections: methicillin-sensitive Staphylococcus aureus (MSSA) was responsible in all cases. All 5 combined infections were due to MSSA. One bacteremia was due to MSSA and one was due to Staphylococcus epidermidis.

Two complicated infections occurred during the study period: a metastatic pulmonary infection during the first period, and a metastatic costovertebral septic arthritis in the second period, respectively. No death related to an infection was observed.

### Infections per patient

We were interested in the number of infectious events per patient. At least one AVF-related infection occurred in only 12 of the 162 included patients; 150 patients never had any AVF infection. During Period 1, three patients had one infection each. During Period 2, one infection was documented in 5 patients and 4 patients had 2 infections, respectively. Recurrence of AVF-related infection was thus observed only during the period 2.

### Zero-inflated Poisson model

We performed a Zero-inflated Poisson regression to test both the link between the type of cannulation and the probability to have no infection, and the link between type of cannulation and the probability to have a recurrent infection. According to this model, the estimate for the excess zero coefficient is not significant (p = 0.9), meaning that the probability to be free from infection does not differ with either RLC or BHC. However, the estimate for the count model coefficient is statistically significant (p = 0.02). This latter result indicates that, in patients with at least one infection, the probability to have a second infection is more important when BHC is used (Table 3).

**Table 3:** ZIP model.

Covariate	Estimate	P value
**Buttonhole cannulation**		
Excess zero coefficient	7,39	0,9
Count model coefficient	2,52	0,02

In order to verify this hypothesis, we conducted a sensitivity analysis (Table 4). We performed two more ZIP models in two different sets of data: data set 1 in which we arbitrary increased the number of all infections to 14 in period 2 (addition of one recurrent infection), and data set 2, in which we arbitrary increased the number of all infections to 17 in period 2 (addition of 4 recurrent infections). In increasing the number of recurrent infection in period 2, we observed an increased significance of the count model coefficient whereas excess zero coefficient was still non significant. Therefore variations of the results seem to strengthen the hypothesis of an association between BHC and recurrence of infection.

**Table 4:** Sensitivity analysis.

	Data set 1		Data set 2	
Covariate	Estimate	P value	Estimate	P value
**Buttonhole cannulation**				
Excess zero coefficient	7,31	0,9	6.32	0.89
Count model coefficient	2,69	0,0005	2,97	7,2.10^-5

Data set 1: arbitrary increase of all infections to 14 in period 2. Data set 2: arbitrary increased of all infections to 17 in period 2.

## Discussion

This is the first study that has, to the best of our knowledge, thoroughly investigated AVF-related infections in a European low-care satellite HD unit. Such infections are rare events. Indeed the incidence rate of all infections in our study is 0.13/1000 and 0.05/1000 AVF-days during Period 2 and 1, respectively. Though untoward outcomes can be observed; we only report two complicated infections in our populations, one in period one and one in period 2.

Labriola and al. [11] showed a significant increase of AVF-related infections in the in-center HD patients of the same Nephrology department as those described in the present study after conversion from RLC to BHC (0.17 vs. 0.43 per 1000 AVF-days, respectively). Careful staff re-education allowed to return the incidence to a rate similar to that of RLC, and to avoid almost completely the occurrence of complicated AVF infections. It should be acknowledged here that satellite units are low-care units as compared to in-center ones. Patients are younger and have less comorbidities than in-center HD patients. Diabetes mellitus is particularly rare in our population. The theoretical risk for overall infections may thus be lower. Moreover, knowledge of the patients’ AVF is an important point for BHC, because angulation and particularities of the track should be respected. Nursing team and number of patients in satellite units are smaller than in in-center HD; this may thus contribute to a better knowledge of each AVF. Interestingly, our team counts only ten nurses, and the turnover of the caregivers is very low.

The overall incidence rate of infection in our satellite unit is also lower than that published for the home HD patients. Muir et al [13] reported an incidence rate of 0.39 infectious events per 1000 AVF-days in home HD patients using BHC, while the group of home HD patients using rope-ladder cannulation had 0.10 infectious events/1000 AVF-days (unadjusted IRR, 3.85; 95% CI, 1.66 to 12.77; P = 0.03). Home HD patients are autonomous patients, without any comorbid condition requiring medical supervision during HD session, thus pretty similar to the patients enrolled in our satellite unit. Therefore we cannot explain the difference observed between Muir’s results and ours by patients’ characteristics. Vascular access use is different between these two populations, as home HD patients perform dialysis more frequently and for longer sessions then patients in satellite unit. The other major difference between satellite dialysis and home HD patients that may potentially explain this discrepancy is nurses’ supervision. BHC requires a strict adherence to the protocol. Rigorous disinfection before and after scab removal is crucial. Scab removal must be complete to prevent scab particles, which contain bacteria, from entering the blood. These hygienic rules may be more carefully applied in satellite units because of the strict surveillance by the nursing staff.

More recently, Wong et al [6] published a systematic review on buttonhole versus rope-ladder cannulation. No difference was found in randomized control trials in cannulation pain, even if pooled observational studies showed a statistical reduction of the pain. This review pointed out to an association between local and systemic infections and BHC technique, for both in-center and home HD patients. Three studies however reported a decrease in infectious complications rate when stricter procedures were used for buttonhole technique [11, 14–15]. However, many variations in cannulation technique existed among studies, and many descriptions of the procedure were incomplete and unclear.

Methodological points should further be considered. AVF-related infections are often described using an incidence rate. However an incidence rate lacks important information as it does not appropriately address the risk of infection recurrence. Indeed, two events in a same patient are considered as two separate events, as it would be for two different subjects in the incidence rate calculation, thus supposing that the events are totally independent. The use of a ZIP model proposes a different approach, by providing two estimates: one for the probability of having “no infection”, and one for the probability of having a “recurrence of an infection”. The power of the results in the present study is very limited by both the small number of patients and events. However, we found a significant zero-inflated estimate and a non significant count model estimate when infection is modelled for by the type of cannulation, meaning that the probability of being free from AVF related infection is not different between rope ladder and buttonhole cannulations techniques, at least in this population. The results of the sensitivity analysis also seem to strengthen our results. By contrast, the main problem with BHC could be the risk of infection recurrence, an event that occurred in 4 of our patients. This implies that emphasis should be put on preventative strategies after a first episode of BH related infection. There are currently no strong recommendations in this matter except for using another cannulation technique. At the time of AVF related infection in our patients, the site of cannulation was not systematically changed, a policy that has recently been modified: we now change the cannulation site in case of suspected local AVF-related infection, even if it is a local infection without systemic symptoms. In case of bacteremia without local signs, cannulation sites are not changed. In addition, we now use prophylactic topical mupirocin in all patients after a first infection [14]. The policy of a systematic switch to the RLC in patients with recurrent BHC related infection, as advocated by some authors [6], appears thus premature, at least in autonomous patients receiving HD in a satellite unit. Finally, future will tell us whether the use of the moist healing technique is beneficial to reduce infection rates in all patients [16].

Another methodological question regards access survival. AVF loss is a competing event when AVF related infection is concerned, as it prevents the observation of the event of interest. One can postulate that a better vascular access survival with BHC can lead to an increase of access’ complications rate. A competing risk model appropriate to this situation should therefore be applied to discriminate between potential benefits of BHC (better access survival) and its related complications, such as infectious events, but this analysis could only be made in large cohorts of patients.

The present study has some limitations, mainly because it is a retrospective analysis. Some interesting covariates that might have influenced outcome were not available. In particular, the impacts of self-cannulation or of the use of Biohole ™ plug during the track creation [17] have been studied. Secondly, the study has got a pre-post design, with some patients participating in the two periods. However, only one of the three patients having an infection in period 1 participated in period 2, without any recurrence of infection. There were an overlap of 15 patients; their characteristics were not different from the other patients. As the risk of infection is more important just after the cannulation switch [11], we think that this overlap would at worst have overestimated the rate of infection in the BHC group, and we decided not to remove them from the analysis. The retrospective design was also a limitation to evaluate other outcomes; cannulation pain, determination of who did the punctions (patients themselves or nurses) and AVF survival could not be studied here. Finally, adjustment on baseline characteristics was not possible because of small numbers in each group and because the event was rare.

In conclusion, BHC is not associated with an increased risk of de novo infection in our HD population from a low-care satellite dialysis unit. In this specific population highly educated about vascular access and closely supervised by nursing team for cannulation process, BHC seems to be a safe technique. The risk of recurrent infections may be the only risk increased with the buttonhole cannulation, a finding not highlighted by previous studies that were based on incidence rate calculation. In the rare patients with AVF infection it is probably necessary to change cannulation sites. Further studies have to identify those patients at risk of recurrence to implement targeted preventative strategies.

## Supporting Information

S1 Dataset Data set used for statistical analysis. (XLSX)
